# Evaluation of the utility of cardiac biomarkers for risk stratification in patients with lower extremity artery disease: A retrospective study

**DOI:** 10.1371/journal.pone.0321491

**Published:** 2025-04-29

**Authors:** Leyla Schweiger, Reinhard B. Raggam, Gabor Toth-Gayor, Philipp Jud, Alexander Avian, Viktoria Nemecz, Katharina Gütl, Marianne Brodmann, Thomas Gary

**Affiliations:** 1 Department of Internal Medicine, Division of Angiology, Medical University of Graz, Graz, Styria, Austria; 2 Department of Internal Medicine, Division of Cardiology, Medical University of Graz, Graz, Styria, Austria; 3 Division of Statistics and Documentation, Institute for Medical Informatics, Medical University of Graz, Graz, Styria, Austria; National Cerebral and Cardiovascular Center: Kokuritsu Junkankibyo Kenkyu Center, JAPAN

## Abstract

Critical limb threatening ischemia (CLTI) is associated with a one-year mortality rate of up to 25% making prompt diagnosis essentially. This study aims to investigate if cardiac biomarkers may serve as an effective tool for risk stratification in patients with lower extremity artery disease (LEAD). For this cross-sectional retrospective analysis, 21712 patients with LEAD were screened for eligibility from 2004 to 2020. Out of these patients, 367 were included and subdivided into those with CLTI and those without CLTI. Cardiac biomarkers, including N-terminal prohormone of brain natriuretic peptide (NT-proBNP), troponin, NT-proBNP/troponin ratio, creatin kinase myocardial band (CK-MB) and myoglobin, were retrospectively analyzed. Fifty-nine patients had CLTI (16.1%) with higher rates of NT-proBNP, NT-proBNP/troponin ratio, CK-MB and myoglobin (all p < 0.05) compared to non-CLTI patients. In univariate analysis, NT-proBNP, NT-proBNP/troponin ratio, CK-MB, myoglobin, age, C-reactive protein and non-insulin dependent diabetes mellitus (NIDDM) were associated with CLTI (all p < 0.05). In multivariate analysis, age and NIDDM remained significant predictors (all p < 0.05) while cardiac biomarkers were not independently associated with CLTI. Troponin, NT-proBNP and myoglobin were associated with mortality in univariate analysis (all p < 0.05). In multivariate analysis, troponin only remains to be associated with mortality (p = 0.001). Selected cardiac biomarkers failed to demonstrate statistically significant differentiation between CLTI and non-CLTI patients with LEAD, while troponin may be potentially associated with mortality.

## 1. Introduction

Peripheral artery disease (PAD) encompasses a wide range of arterial disorders affecting both the upper and lower extremities, as well as arteries in the carotid, vertebral, mesenteric, and renal regions, primarily caused due to atherosclerosis [1]. One predominant manifestation of PAD is lower extremity artery disease (LEAD), which exhibits a prevalence ranging from 5–8% affecting estimated more than 200 million individuals worldwide [2]. LEAD is associated with a significant mortality and morbidity, particularly in advanced stages such as chronic limb-threatening ischemia (CLTI). CLTI is characterized by ischemic rest pain or by tissue damage including infections, ulcers and necrosis of the lower limb with a reported one-year mortality rate of 25% and a one-year amputation rate of 30% [1,3]. Consequently, early diagnosis and adequate treatment strategies for CLTI are essential to prevent further complications.

LEAD frequently coexists with other arterial disorders, including cerebrovascular (CVAD) and coronary artery disease (CAD). The prevalence of LEAD among patients with acute coronary syndrome (ACS) is estimated by 40% and LEAD is an independent predictor of subsequent cardiovascular events [4,5]. In the Reach Registries, more than 50% of the patients with LEAD have concomitant CAD and/or CVAD [6]. While cardiac biomarkers like N-terminal prohormone of brain natriuretic peptide (NT-pro-BNP) or troponin have traditionally been studied in the context of cardiac diseases, recent investigations have evaluated their relevance also in LEAD, potentially due to a high comorbidity of different atherosclerotic disorders in those patients. Troponin and NT-pro-BNP have emerged as promising prognostic indicators for LEAD in several studies. It has been shown that these two parameters are associated independently with the incidence of LEAD. Additionally, it has been demonstrated that elevated troponin T levels are associated with higher mortality and amputation rates [2,7–10]. Less commonly investigated cardiac biomarkers, like NT-proBNP/troponin ratio, have additionally been studied for differentiation of ACS and whether troponin elevation in the emergency department was cardiac-related or not. This ratio may be helpful to discriminate ACS from Takotsubo cardiomyopathy and if troponin elevation was caused from conditions other than ACS [4,11]. Since 34% of CLTI patients present with elevated troponin levels, we want to investigate if different cardiac biomarkers may serve as prognostic markers and mortality predictors in patients with LEAD, who underwent endovascular revascularization, and may be useful in the future for risk stratification in these patients [8].

## 2. Materials and methods

### 2.1. Study design and patient cohort

This study is a retrospective cross-sectional analysis, wherein a blinded, pseudonymized dataset of patients with known LEAD were analyzed. A comprehensive screening of patients, who had been admitted to the outpatient clinic of Angiology of the Medical University of Graz due to LEAD from 2004 to 2020, was performed on 01.04.2022 via a fully electronic patient information system, called Medical Documentation and Communication network of Styria (MEDOCS). MEDOCS is installed in the province of Styria, Austria, to provide electronic health data from all public Styrian hospitals and hospital alliances [12]. Patient’s demographics, clinical parameters, comorbidities, and laboratory findings were recorded and analyzed. During the admission at the outpatient clinic of Angiology of the Medical University of Graz, each patient had undergone a detailed medical history including clinical symptoms and physical examination. Additionally, measurement of an ankle-brachial index (ABI), a duplex ultrasonography of the lower leg arteries, and obtainment of blood samples had been performed.

Inclusion criteria for this study were a diagnosed LEAD with the requirement of an endovascular intervention and available laboratory results, including cardiac biomarkers, which had been obtained within eight days prior to the endovascular intervention. Patients were excluded from this study if no endovascular intervention was necessary or if laboratory parameters were either not available or older than eight days prior to the endovascular intervention. All eligible patients were further subdivided into patients with and without CLTI. According to the recent guidelines, CLTI was defined as LEAD characterized by ischemic rest pain, with or without tissue loss or infection according to Fontaine classification stage III and IV [13].

Cardiac biomarkers were defined as troponin, NT-proBNP, NT-proBNP/troponin ratio, creatin kinase myocardial band (CK-MB) and myoglobin and were measured via a lithium heparin tube by routine laboratory work-up. For the detection of troponin and NT-proBNP, the fully automated Cobas 8000 test system (Roche Diagnostics) was used according the manufacturer’s instructions. Plasma samples were processed after centrifugation within 60–90min. Briefly, troponin was measured in plasma samples by using the Elecsys® Troponin-T high sensitive electrochemiluminescence immunoassay (Roche Diagnostics) with a detection limit of 3ng/L, a normal reference range from 3–14ng/L and a measuring range of 3–10.000ng/L. NT-proBNP was measured in plasma samples by using the Elecsys® NT-proBNP II electrochemiluminescence immunoassay (Roche Diagnostics), showing a limit of detection of 5pg/mL with a normal reference range from 5–125pg/mL and a measuring range of 5–30.000pg/mL. Both assays were routinely controlled using PreciControl Cardiac II for the various troponin and NT-proBNP concentration ranges; quality controls were run standardized at least once within 24 hours. There were changes in the assays over the 16-year study period. However, to ensure consistency throughout the entire duration of the study, we consistently applied the same thresholds for interpretation of the biomarker results.

The data regarding mortality, extracted from the MEDOCS system, represents all-cause mortality, which was consistently used throughout the 16-year study period.

### 2.2. Statistical analysis

Data are given as median and interquartile range (IQR) for continuous data, and as a frequency for categorical data. Univariate logistic regression analysis was performed to identify potential predictors for CLTI and mortality. If distribution of predictors did not lead to normally distributed residuals, the initial predictor was transformed (e.g., logarithmized). All variables showing an univariate p-value <0.05 were analyzed regarding multicollinearity and excluded if necessary. For the final set of potential predictors, multivariate logistic regression was performed using backwards selection to identify independent predictors. Odds ratio (OR) with 95% confidence intervals (95% CI) were calculated. A p-value less than 5% was considered significant. For data analysis, IBM SPSS Statistics 26 (IBM Corporation, Armonk, NY, USA) was used.

### 2.3. Ethical statement

The study was approved by the Institutional Review Board of the Medical University Graz, Austria (EK 34–006 ex 21/22). Due to the retrospective nature of the study, it was not necessary to obtain informed consent from the patients according to the Institutional Review Board.

## 3. Results

In total, 21712 patients with LEAD were screened for eligibility. Of those, 367 patients fulfilled all inclusion criteria, had available cardiac biomarkers and were considered for the final analysis. Fifty-nine patients (16.1%) had CLTI. Patients’ baseline characteristics are presented in Table 1.

**Table 1:** Patients’ baseline characteristics.

Total patients, n	367
Age (years), median (IQR)	71 (62-80)
Sex, n (%)	
Male	226 (61.6)
Female	141 (38.4)
Fontaine Stage, n (%)	
II	308 (83.9)
III	10 (2.7)
IV	49 (13.4)
Smoking, n (%)	177 (48.2)
BMI, median (IQR)	26.4 (23.8-29.6)
Comorbidity, n (%)	
Obesity	204 (55.6)
Arterial Hypertension	309 (84.2)
Diabetes	143 (39.0)
IDDM	44 (12.0)
NIDDM	99 (27.0)
CAD	78 (21.3)
CVAD	75 (20.4)
Visceral artery disease	4 (1.1)
Renal artery disease	6 (1.6)
Comedication, n (%)	
Statins	151 (41.1)
Antiplatelet therapy	353 (96.2)
Mortality, n (%)	53 (14.4)
Cardiac biomarkers, median (IQR)	
Troponin	
≤0.005 (ng/mL), n (%)	289 (78.7)
>0.005 (ng/mL), n (%)	78 (21.3)
NT-proBNP (pg/mL)	363 (131-1448)
NT-proBNP/troponin ratio	58600 (23600-146400)
CK-MB (U/L)	14 (11-19)
Myoglobin (ng/mL)	48.3 (35.7-75.7)

Abbreviations: BMI: body mass index; CAD: coronary artery disease; CK-MB: creatin kinase myocardial band; CVAD: cerebrovascular artery disease; IDDM: insulin dependent diabetes mellitus; IQR: interquartile range; NIDDM: non-insulin dependent diabetes mellitus NT-proBNP: N-terminal prohormone of brain natriuretic peptide.

**Fig 1:**
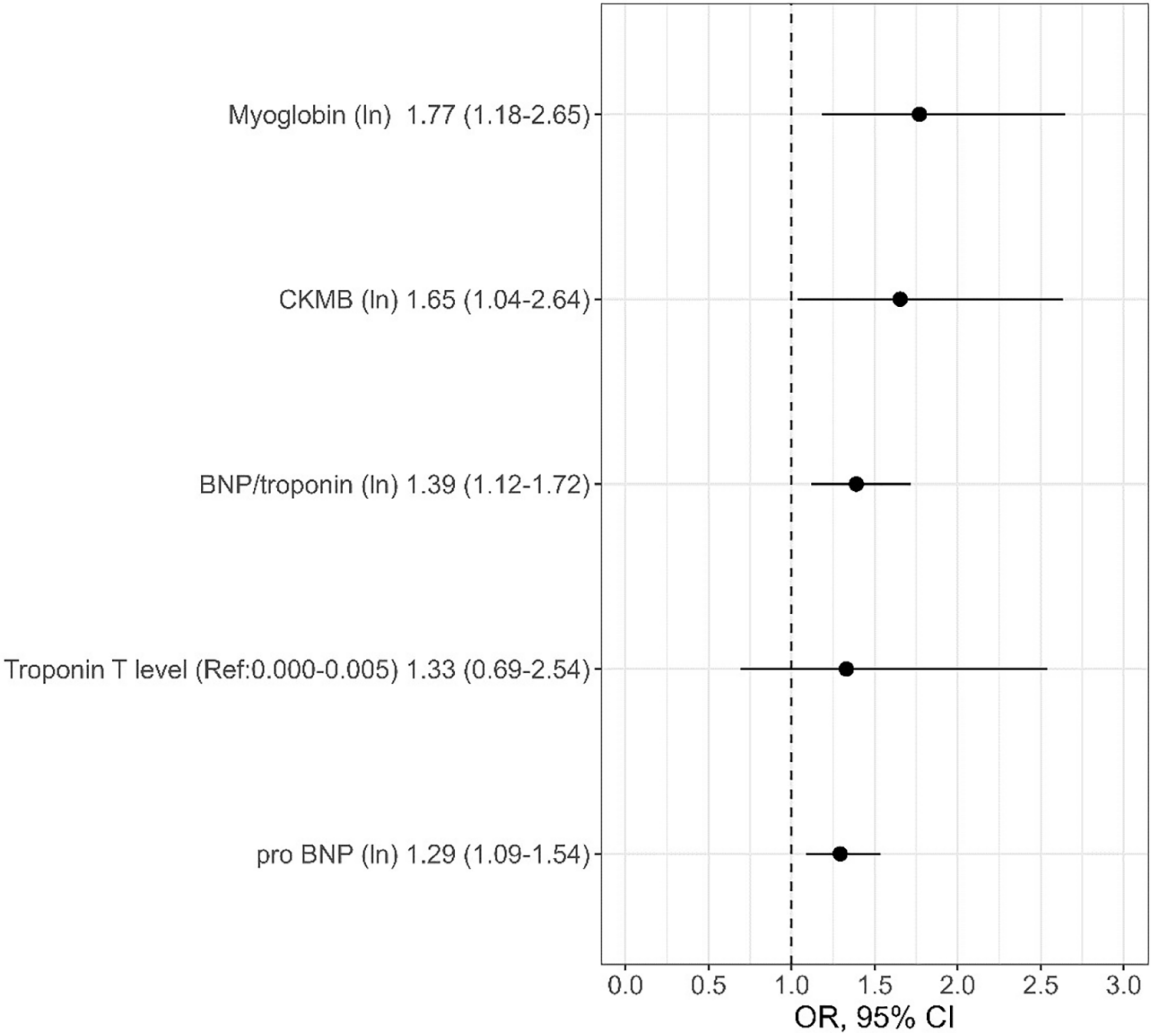
Univariate analysis of cardiac biomarkers regarding discrimination between CLTI and non-CLTI.

Between the subgroup with CLTI and without CLTI in univariate analysis, all cardiac biomarkers except troponin were significantly elevated in patients with CLTI (all p < 0.05) (Fig 1). Additionally, patients with CLTI were in univariate analysis significantly older (p < 0.001), were less commonly smokers (p = 0.018), had less commonly a non-insulin dependent diabetes mellitus (NIDDM) (p = 0.006) and a lower body mass index (BMI) (p = 0.002). Male/female ratio as well as other previous cardiovascular comorbidities and comedications were comparable between both groups. In multivariate analysis, age (OR: 1.06, 95%CI: 1.03–1.10) and NIDDM (OR: 0.22, 95%CI: 0.09–0.55) remained significant predictors for CLTI. Cardiac biomarkers, however, failed to be significant (Table 2).

**Table 2:** Uni- and multivariate analysis of clinical and laboratory parameters between patients with CLTI and without CLTI.

	CLTI (n = 59)	Non-CLTI (n = 308)	Univariate			Multivariate		
			OR	95% CI	p-value	OR	95% CI	p-value
Age (years), median (IQR)	78 (68-85)	70 (61-79)	1.06	1.03-1.09	**<0.001**	1.06	1.03-1.10	**<0.001**
Male sex, n (%)	37 (62.7)	189 (61.4)	0.94	0.53-1.68	0.845			
Smoking, n (%)	20 (33.9)	157 (51.0)	0.49	0.28-0.88	**0.018**			
BMI, median (IQR)	24.9 (21.6-28.1)	26.9 (23.9-29.9)	0.89	0.83-0.96	**0.002**			
Comorbidity, n (%)								
Obesity	26 (44.1)	178 (57.8)	0.58	0.33-1.01	0.054			
IDDM	8 (13.6)	36 (11.7)	1.19	0.52-2.70	0.686			
NIDDM	7 (11.9)	92 (29.9)	0.32	0.14-0.72	**0.006**	0.22	0.09-0.55	**0.001**
CAD	12 (20.3)	66 (21.4)	0.94	0.47-1.87	0.851			
CVAD	11 (18.6)	64 (20.8)	0.87	0.43-1.78	0.710			
Visceral artery disease	1 (1.7)	3 (1.0)	-*	-*	-*			
Renal artery disease	0 (0.0)	6 (1.9)	-*	-*	-*			
Comedication, n (%)								
Statins	22 (37.3)	129 (41.9)	0.83	0.47-1.47	0.512			
Antiplatelet therapy	56 (94.9)	297 (96.4)	0.96	0.53-1.76	0.918			
Cardiac biomarkers, median (IQR)								
Troponin (ng/mL)			1.33	0.69-2.54	0.394			
≤0.005, n (%)	44 (74.6)	245 (79.5)						
>0.005, n (%)	15 (25.4)	63 (20.5)						
NT-proBNP (pg/mL)	1031 (177-2379)	310 (127-1085)	1.29	1.09-1.54	**0.004**			
NT-proBNP/troponin ratio	116800 (23800-296800)	53000 (23600-127284)	1.39	1.12-1.72	**0.003**			
CK-MB (U/L)	16 (12-20)	14 (10-19)	1.65	1.04-2.64	**0.034**			
Myoglobin (ng/mL)	55.1 (42.9-108.6)	46.8 (34.6-74.5)	1.77	1.18-2.65	**0.005**			

Abbreviations: BMI: body mass index; CAD: coronary artery disease; CK-MB: creatin kinase myocardial band; CVAD: cerebrovascular artery disease; IDDM: insulin dependent diabetes mellitus; IQR: interquartile range; NIDDM: non-insulin dependent diabetes mellitus; NT-proBNP: N-terminal prohormone of brain natriuretic peptide.

*: No adequate statistical analysis was made due to a too low number.

In univariate analysis regarding mortality, troponin, NT-proBNP and myoglobin were significant predictors (all p < 0.05). In multivariate analysis, only troponin remained a significant predictor for mortality (OR: 2.96, 95%CI: 1.59–5.5) (Table 3).

**Table 3:** Uni- and multivariate analysis of cardiac biomarkers regarding mortality.

	Univariate analysis		Multivariate analysis	
	OR (95% CI)	p-value	OR (95% CI)	p-value
Troponin	2.96 (1.59-5.50)	**0.001**	2.96 (1.59-5.50)	**0.001**
NTproBNP	1.26 (1.05-1.50)	**0.012**		
NT-proBNP/troponin ratio	1.02 (0.82-1.27)	0.842		
CK-MB	0.89 (0.56-1.40)	0.613		
Myoglobin	1.87 (1.23-2.83)	**0.003**		

Abbreviations: CK-MB: creatin kinase myocardial band; NT-proBNP: N-terminal prohormone of brain natriuretic peptide.

## 4. Discussion

This retrospective analysis could demonstrate that cardiac biomarkers do not seem to be robust for the prediction of CLTI in patients with LEAD undergoing endovascular interventions. Although most patients’ characteristics were comparable to previous studies, there were also some differences between patients with CLTI and without CLTI in this study. The average age of our patient cohort was in line with findings from prior studies supporting the association between LEAD and advanced age by which a prevalence of about 20% in patients older than 70 years has been described [13,14]. Interestingly, there was a comparatively lower prevalence of CAD in the investigated cohort, with only 21% of patients having a documented history of CAD. It has been reported that 30% of patients undergoing peripheral vascular surgery have a concomitant CAD and that 52% of patients with symptomatic LEAD had concomitant CAD [15,16]. In our entire cohort, approximately 39% had a documented diabetes mellitus, with 12% of our patients having insulin-dependent diabetes mellitus (IDDM) and 27% having NIDDM. The literature suggests that about 20–30% of LEAD patients have diabetes mellitus as a comorbidity [17]. This number is slightly lower than in our patient population. Nearly half of our patients were smokers, which is again comparable to available literature [18]. Overall, our total patient cohort seems to be similar to previous studies.

Comparisons between our CLTI and non-CLTI group revealed some differences to existing data. In our cohort, about 16% of LEAD patients had CLTI, which is similar to the reported data of recent literature, where the prevalence of CLTI among LEAD patients is reported to be 11% [19]. Regarding gender distribution between CLTI and non-CLTI patients, more men were in both groups, although this difference was marginal and statistically not significant. Based on the available data, however, we might have expected to find more women in the CLTI group, as they tend to present later and more often with an advanced LEAD stage [20]. Despite similar gender distributions, the CLTI group exhibited a significantly higher average age and age was confirmed as a potential independent risk factor for CLTI in multivariate analysis confirming that age may play a pivotal role in disease severity and progression. This finding is consistent with the existing literature indicating that the likelihood of CLTI in patients over 80 years of age is 2–3 times higher than in younger patients [21]. Moreover, this emphasizes also the importance of age in predicting disease severity and the necessity for targeted interventions in older populations. The BMI in our cohort was statistically lower in the CLTI group compared to the non-CLTI group. This may be explained by the previously described ‘obesity paradox’. In this phenomenon, a higher BMI is associated with a more favorable prognosis regarding cardiovascular diseases [22]. The statistically significant lower prevalence of smokers in our CLTI group is, however, in complete contrast to the existing literature since smoking is the main risk factor for LEAD, and active nicotine abuse is associated with a 2–3-fold increased risk of LEAD [23]. Additionally, lower rates of concomitant NIDDM were observed in the group of CLTI patients, which is again contrary to previous studies as several data demonstrated that diabetes mellitus worsens the outcome for LEAD and is associated with higher amputation rates [24,25]. One explanation for the lower prevalence rates of smokers in the CLTI group would be that the recent analysis did not include ex-smokers. Due to the inclusion criteria of patients with cardiac biomarkers, we likely have a high proportion of patients with a cardiovascular history, who may have already quit smoking. From our cohort according to the guidelines, each patient is recommended to join a prevention program, including smoking cessation, before revascularization is indicated. However, even with intensive programs, the rates of nicotine abstinence are reported to be at maximum 21.3% [26]. Furthermore, one study described the paradox of reduced restenosis rates of after lower limb endovascular revascularization in people smoking 10 cigarettes and more [27]. Our study did not discriminate if patients had undergone a previous endovascular recanalization or not. Therefore, it may be possible that patients with an initial higher LEAD stage had undergone previously to study inclusion an endovascular recanalization, which had led to an amelioration of their symptoms and subsequently to a decrease of their LEAD stage. Those patients who continued smoking afterwards may develop reduced restenosis rates according t Schillinger et al [27] and it may be assumed also a lower symptomatic LEAD stage. The lower rates of NIDDM in patients with CLTI may be explained by the fact that those patients presenting with CLTI do not know about their concomitant NIDDM due to a neglected self-health responsibility. This finding may not accurately depict the health status of individuals with NIDDM. Rather, it reflects the contrasting severity of the condition between those with IDDM and those without diabetes mellitus. In the CLTI cohort of Darling et al [28], the number of NIDDM was lower than the number of IDDM or non-diabetic patients. Additionally, the long-term mortality and the adverse event rate was lower in the NIDDM group, like in our cohort. Moreover, it should be noted that our analysis only included selected patients who underwent endovascular interventions. Those who were primarily treated with vascular surgery or required immediate amputation due to complex morphological vascular changes were not included in the evaluation. It is important to note that in this retrospective study, diabetes mellitus was identified based on pre-existing diagnoses rather than on HbA1c values. This approach could have led to underdiagnosis of diabetes mellitus. Another difference in the prevalence of smoking and NIDDM in the CLTI group could be attributed to a selection bias as the study did not included the most severely affected patients with acute ischemia leading to acute amputation or other vascular surgery procedure.

Regarding cardiac biomarkers, NT-proBNP levels were significantly elevated in the CLTI group in univariate analysis but failed to be an independent predictor for CLTI in multivariate analysis. This is contrary to the study by Kumakura et al. [29], in which over 800 patients were included. This study reported that elevated NT-proBNP levels were independently associated with CLTI. Potential reasons for the discrepancy between our results and the result of Kumakura et al. [29] may be explained by the larger sample size, the prospective study design and routine collection of NT-proBNP. This could explain why NT-proBNP cannot be used to differentiate independently between CLTI and non-CLTI in our cohort. Similarly, NT-proBNP/troponin ratio, CK-MB and myoglobin achieved significance only in the univariate analysis, but failed in multivariate analysis suggesting a nuanced role in predicting CLTI. Additionally, troponin failed to demonstrate a statistically significant difference between the groups in univariate analysis, prompting considerations about the choice of troponin assay and its implications for diagnostic accuracy. Hicks et al. [30] were also unable to find a strong association of troponin and LEAD among their cohort. Overall, the respective cardiac parameters did not appear to be predict robustly and independently CLTI in patients with LEAD. However, compared to other studies, our study differentiated between patients with CLTI and non-CLTI. Most previous studies used the ankle brachial index (ABI) for the diagnosis of LEAD, while we have included only patients who had undergone an endovascular recanalization due to their symptomatic LEAD. Therefore, we did not include early stage of LEAD. Whether these parameters, in combination with others, can help identify LEAD patients who require urgent revascularization and aggressive conservative management remains to be clarified. It may be worthwhile to consider differentiation based on earlier stages (Fontaine stages I and IIa) as well.

In univariate analysis, troponin, NT-proBNP and myoglobin were statistically significant associated with mortality. This finding is comparable to the existing literature, wherein troponin and NT-proBNP is associated with increased mortality in patients with LEAD [10,31]. In multivariate analysis, only troponin remains to be associated with mortality, which is consistent with the study by Linenmann et al. [7] in which troponin was also related with higher mortality and amputation rates. On the other side, our multivariate models did not include additional confounding factors, which may influence the association results including arterial hypertension, renal insufficiency. Due to the fact that only a small number of patients were included and therefore the multivariate models may be at risk of overfitting, larger cohorts are needed to validate our results on mortality and also on CLTI.

Limitations of our study are the retrospective design and reliance on data exclusively from medical records. The use of medical records as the primary data source may lead to potential limitations to the study. The study only includes patients who underwent endovascular interventions, excluding those who received surgical treatment or immediate amputation. This represents a selection bias and may limit the applicability of the findings to the broader LEAD population. Additionally NT-proBNP and troponin were not routinely collected in all patients, potentially skewing the results. This selective inclusion may have biased the findings toward patients with more severe cardiovascular comorbidities. Due to inclusion of only patients with available cardiac biomarker data and those undergoing endovascular interventions, the results of this study cannot be reproduced on the general LEAD population, as those LEAD patients with a less severe disease or those treated surgically were excluded. Furthermore, our small sample size of patients with CLTI reduces also the statistical power and limits the robustness of conclusion. Additional selection bias are that no information about the proportion of former smokers were recorded.

## 5. Conclusion

In conclusion, while cardiac biomarkers tend to be elevated in patients with CLTI, our study did not find them to be significantly indicative of CLTI presence in our cohort. This underscores on the one hand the complex nature of risk factors in LEAD and, on the other hand, these findings may be accompanied with methodological limitations. Future research should focus on larger sample sizes and more diverse cohorts to thoroughly evaluate the utility of these biomarkers for risk stratification in LEAD patients.

## Supporting information

S1 Data Min data set. (XLSX)

## References

[1] Aboyans V, Ricco JB, Bartelink MEL, Björck M, Brodmann M, Cohnert T, et al. 2017 ESC Guidelines on the Diagnosis and Treatment of Peripheral Arterial Diseases, in collaboration with the European Society for Vascular Surgery (ESVS): Document covering atherosclerotic disease of extracranial carotid and vertebral, mesenteric, renal, upper and lower extremity arteries Endorsed by: the European Stroke Organization (ESO)The Task Force for the Diagnosis and Treatment of Peripheral Arterial Diseases of the European Society of Cardiology (ESC) and of the European Society for Vascular Surgery (ESVS). Eur Heart J. 2018 Mar 1;39(9):763–816. 28886620 10.1093/eurheartj/ehx095

[2] Fowkes FG, Rudan D, Rudan I, Aboyans V, Denenberg JO, McDermott MM, et al. Comparison of global estimates of prevalence and risk factors for peripheral artery disease in 2000 and 2010: a systematic review and analysis. Lancet. 2013 Oct 19; 382(9901):1329–40. 23915883 10.1016/S0140-6736(13)61249-0

[3] Norgren L, Hiatt WR, Dormandy JA, Nehler MR, Harris KA, Fowkes FG, et al. Inter-Society Consensus for the Management of Peripheral Arterial Disease (TASC II). Eur J Vasc Endovasc Surg. 2007;33(Suppl 1):S1–75. 17140820 10.1016/j.ejvs.2006.09.024

[4] Kim DH, Lee SH, Kim SC, Kim T, Kang C, Jeong JH, et al. The ratio of N-terminal pro-B-type natriuretic peptide to troponin I for differentiating acute coronary syndrome. Am J Emerg Med. 2019;37(6):1013–1019. doi: 10.1016/j.ajem.2018.08.035 30122508

[5] Ankle Brachial Index Collaboration, Fowkes FG, Murray GD, Butcher I, Heald CL, Lee RJ, et al. Ankle brachial index combined with Framingham Risk Score to predict cardiovascular events and mortality: a meta-analysis. JAMA. 2008 Jul 9;300(2):197-208. 18612117 10.1001/jama.300.2.197 PMC2932628

[6] Bauersachs R, Zeymer U, Brière JB, Marre C, Bowrin K, Huelsebeck M. Burden of Coronary Artery Disease and Peripheral Artery Disease: A Literature Review. Cardiovasc Ther. 2019 Nov 26;2019:8295054. 32099582 10.1155/2019/8295054 PMC7024142

[7] Linnemann B, Sutter T, Herrmann E, Sixt S, Rastan A, Schwarzwaelder U, et al. Elevated cardiac troponin T is associated with higher mortality and amputation rates in patients with peripheral arterial disease. J Am Coll Cardiol. 2014 Apr 22;63(15):1529–38. 23792625 10.1016/j.jacc.2013.05.059

[8] Sarveswaran J, Ikponmwosa A, Asthana S. Should cardiac troponins be used as a risk stratification tool for patients with chronic critical limb ischaemia?. Eur J Vasc Endovasc Surg. 2007;33(6):703–707. doi: 10.1016/j.ejvs.2006.11.041 17275360

[9] Matsushita K, Kwak L, Yang C, Pang Y, Ballew SH, Sang Y, et al. High-sensitivity cardiac troponin and natriuretic peptide with risk of lower-extremity peripheral artery disease: the Atherosclerosis Risk in Communities (ARIC) Study. Eur Heart J. 2018 39(25):2412–9. 29579246 10.1093/eurheartj/ehy106 PMC6031056

[10] Clemens RK, Annema W, Baumann F, Roth-Zetzsche S, Seifert B, von Eckardstein A, et al. Cardiac biomarkers but not measures of vascular atherosclerosis predict mortality in patients with peripheral artery disease. Clin Chim Acta. 2019;495:215–220. doi: 10.1016/j.cca.2019.04.061 30981846

[11] Fröhlich G, Schoch B, Schmid F, Keller P, Sudano I, Lüscher TF, et al. Takotsubo cardiomyopathy has a unique cardiac biomarker profile: NT-proBNP/myoglobin and NT-proBNP/troponin T ratios for the differential diagnosis of acute coronary syndromes and stress induced cardiomyopathy. Int J Cardiol. 2012 Feb 9;154(3):328–32. 22044675 10.1016/j.ijcard.2011.09.077

[12] Gell G, Madjaric M, Leodolter W. HIS purchase projects in public hospitals of Styria, Austria. Int J Med Inform. 2000;58–59:147–155. doi: 10.1016/s1386-5056(00)00083-6 10978917

[13] Horváth L, Németh N, Fehér G, Kívés Z, Endrei D, Boncz I. Epidemiology of Peripheral Artery Disease: Narrative Review. Life (Basel). 2022 Jul 12;12(7):1041 35888129 10.3390/life12071041 PMC9320565

[14] Dua A, Lee CJ. Epidemiology of Peripheral Arterial Disease and Critical Limb Ischemia. Tech Vasc Interv Radiol. 2016;19(2):91–5. doi: 10.1053/j.tvir.2016.04.001 27423989

[15] Hertzer NR, Beven EG, Young JR, O’Hara PJ, Ruschhaupt WF, Graor RA, et al. Coronary artery disease in peripheral vascular patients. A classification of 1000 coronary angiograms and results of surgical management. Ann Surg. 1984;199(2):223–233. doi: 10.1097/00000658-198402000-00016 6696538 PMC1353337

[16] Chen DC, Singh GD, Armstrong EJ. Long-Term Comparative Outcomes of Patients With Peripheral Artery Disease With and Without Concomitant Coronary Artery Disease. Am J Cardiol. 2017 Apr 15;119(8):1146–1152. doi: 10.1016/j.amjcard.2016.12.023 28259239

[17] Marso SP, Hiatt WR. Peripheral arterial disease in patients with diabetes. J Am Coll Cardiol. 2006 Mar 7;47(5):921–9. doi: 10.1016/j.jacc.2005.09.065 16516072

[18] Joosten MM, Pai JK, Bertoia ML, Rimm EB, Spiegelman D, Mittleman MA, et al. Associations between conventional cardiovascular risk factors and risk of peripheral artery disease in men. JAMA. 2012 Oct 24;308(16):1660–7. 23093164 10.1001/jama.2012.13415 PMC3733106

[19] Nehler MR, Duval S, Diao L, Annex BH, Hiatt WR, Rogers K, et al. Epidemiology of peripheral arterial disease and critical limb ischemia in an insured national population. J Vasc Surg. 2014 Sep;60(3):686-95.e2. doi: 10.1016/j.jvs.2014.03.290 24820900

[20] Schramm K, Rochon PJ. Gender Differences in Peripheral Vascular Disease. Semin Intervent Radiol. 2018;35(1):9–16. doi: 10.1055/s-0038-1636515 29628610 PMC5886764

[21] Jude EB, Oyibo SO, Chalmers N, Boulton AJ. Peripheral arterial disease in diabetic and nondiabetic patients: a comparison of severity and outcome. Diabetes Care. 2001;24(8):1433–7. doi: 10.2337/diacare.24.8.1433 11473082

[22] Lavie CJ, McAuley PA, Church TS, Milani RV, Blair SN. Obesity and cardiovascular diseases: implications regarding fitness, fatness, and severity in the obesity paradox. J Am Coll Cardiol. 2014 Apr 15;63(14):1345–54. 24530666 10.1016/j.jacc.2014.01.022

[23] Ding N, Sang Y, Chen J, Ballew SH, Kalbaugh CA, Salameh MJ, et al. Cigarette Smoking, Smoking Cessation, and Long-Term Risk of 3 Major Atherosclerotic Diseases. J Am Coll Cardiol. 2019 Jul 30;74(4):498–507. 31345423 10.1016/j.jacc.2019.05.049 PMC6662625

[24] Duff S, Mafilios MS, Bhounsule P, Hasegawa JT. The burden of critical limb ischemia: a review of recent literature. Vasc Health Risk Manag. 2019 Jul 1;15:187–208. 31308682 10.2147/VHRM.S209241 PMC6617560

[25] Ding N, Kwak L, Ballew SH, et al. Traditional and nontraditional glycemic markers and risk of peripheral artery disease: The Atherosclerosis Risk in Communities (ARIC) study. Atherosclerosis. 2018;274:86–93. doi: 10.1016/j.atherosclerosis.2018.04.042 29753232 PMC5999570

[26] Behrooz L, Abumoawad A, Rizvi SHM, Hamburg NM. A modern day perspective on smoking in peripheral artery disease. Front Cardiovasc Med. 2023 Apr 28;10:1154708. doi: 10.3389/fcvm.2023.1154708 ; PMCID: PMC10175606 37187787 PMC10175606

[27] Schillinger M, Exner M, Mlekusch W, Haumer M, Sabeti S, Ahmadi R, et al. Effect of smoking on restenosis during the 1st year after lower-limb endovascular interventions. Radiology. 2004;231(3):831–8. doi: 10.1148/radiol.2313031088 15163820

[28] Darling JD, Bodewes TCF, Deery SE, Guzman RJ, Wyers MC, Hamdan AD, et al. Outcomes after first-time lower extremity revascularization for chronic limb-threatening ischemia between patients with and without diabetes. J Vasc Surg. 2018 Apr; 67(4):1159–69. 28947228 10.1016/j.jvs.2017.06.119 PMC5862717

[29] Kumakura H, Kanai H, Araki Y, Hojo Y, Kasama S, Sumino H, et al. Differences in brain natriuretic peptide and other factors between Japanese peripheral arterial disease patients with critical limb ischemia and intermittent claudication. J Atheroscler Thromb. 2013;20(11):798–806. doi: 10.5551/jat.18929 23831586

[30] Hicks CW, Wang D, McDermott K, Matsushita K, Tang O, Echouffo-Tcheugui JB, et al. Associations of Cardiac Biomarkers With Peripheral Artery Disease and Peripheral Neuropathy in US Adults Without Prevalent Cardiovascular Disease. Arterioscler Thromb Vasc Biol. 2023 Aug;43(8):1583–91. doi: 10.1161/ATVBAHA.122.318774 37317848 PMC10526698

[31] Kremers B, Wübbeke L, Mees B, Ten Cate H, Spronk H, Ten Cate-Hoek A. Plasma Biomarkers to Predict Cardiovascular Outcome in Patients With Peripheral Artery Disease: A Systematic Review and Meta-Analysis. Arterioscler Thromb Vasc Biol. 2020 Sep;40(9):2018–32. 32640905 10.1161/ATVBAHA.120.314774 PMC7447177

